# Diverging Elevational Patterns of Tree vs. Epiphyte Species Density, Beta Diversity, and Biomass in a Tropical Dry Forest

**DOI:** 10.3390/plants13182555

**Published:** 2024-09-11

**Authors:** Florian A. Werner, Jürgen Homeier

**Affiliations:** 1 Functional Ecology, Institute of Biology and Environmental Sciences, University of Oldenburg, Carl-von-Ossietzkystraße 9-11, 26111 Oldenburg, Germany; 2 Faculty of Resource Management, HAWK University of Applied Sciences and Arts, Daimlerstraße 2, 37075 Göttingen, Germany; 3 Plant Ecology, Georg-August University of Göttingen, 37073 Göttingen, Germany

**Keywords:** alpha diversity, beta diversity, biomass, biotic interactions, competition, determinants of nestedness, Ecuador, species richness, species turnover, Tumbes–Piura dry forest

## Abstract

There is evidence to suggest that vascular epiphytes experience low competition for resources (light, water, and nutrients) compared to terrestrial plants. We tested the hypothesis that low resource competition may lead to higher nestedness among vascular epiphyte assemblages compared to trees. We studied the species composition and biomass of epiphytes and trees along an elevation gradient in a tropical dry forest in SW Ecuador. Both life-forms were inventoried on 25 plots of 400 m^2^ across five elevation levels (550–1250 m). Tree species density and total species richness increased with elevation, whereas basal area and biomass did not show significant trends. Epiphyte species density and richness both increased strongly with elevation, in parallel to biomass. Plot-level compositional changes were similarly strong for both life-forms. We attribute elevational increases in the species richness of trees and epiphytes to increasing humidity, i.e., more mesic growth conditions. We attribute the more pronounced elevational increase in epiphyte biomass, species density, and richness—the latter coupled with a higher degree of nestedness—to the greater moisture dependency of epiphytes and relatively low direct competition for resources. Our study provides a first comparison of elevational trends in epiphyte and tree diversity and biomass for a tropical dry forest.

## 1. Introduction

Rates of tropical forest loss remain high. Understanding elevational trends in biodiversity and their synergies and trade-offs with environmental services, especially carbon storage, is essential for prioritising biodiversity conservation efforts [1,2]. Moreover, climate change impacts are projected to increase strongly, likely to match land-use change as the main threat to biodiversity in the course of the coming decades [3]. Steep elevational gradients constitute the best natural study models available for predicting how vegetation and species assemblages may change due to global climate change [4].

Meanwhile, tropical dry forests remain poorly studied, in part due to the paucity of intact gradients suitable and available for study. Throughout the tropics, dry forest environments have long been preferred for agricultural use and have undergone intense settlement, conversion, and fragmentation [5]. Man-lit fires escape more easily in arid environments, further reducing and degrading natural vegetation. Today, tropical dry forest environments are among the most threatened, degraded and pressured [6]. As a consequence, few mountains in arid or semiarid environments continue to support contiguous gradients of natural vegetation sufficiently intact for ecological studies [7,8,9].

Lowland tropical dry forests tend to be markedly less speciose in trees than moist and wet forests, although this varies greatly among regions and with spatial scale [10]. This trend is mirrored by biomass [11]. Elevational patterns also diverge: in moist and wet tropical environments, tree diversity and biomass usually decrease with elevation, e.g., [12,13]. In contrast, in semi-humid and semi-arid environments, tree diversity can show mid-elevation peaks. This pattern has been attributed to increasing air humidity, decreasing vapour pressure deficit (VPD), and hence lower drought stress towards higher elevations, creating more mesic conditions suitable for a wider variety of taxa [14].

Due to their disconnect from soil water, epiphytes are considered particularly sensitive to measures of desiccation stress such as humidity and VPD [15,16,17]. While vascular epiphytes have developed a wealth of adaptations to avoid desiccation, desiccation tolerance is rare. Hence, few epiphytes are adapted to withstand prolonged periods of drought [18], especially during establishment [19,20]. This sensitivity of vascular epiphytes to water availability is closely reflected by geographical patterns of species diversity. For example, in coastal Ecuador, [21] found that the number of tree species in plots of 0.1 ha decreased moderately from wet forest (114 species) to dry forest (48), while the number of vascular epiphytes decreased dramatically from 127 to just 3. Regarding epiphytic biomass, the highest values have likewise been found in wet mountain environments [22]. However, our understanding of biomass patterns is confined by diverging methodologies, numerous site characteristics, a pronounced bias of studies towards cloud forest sites with exuberant epiphytic vegetation, and the paucity of studies from xeric environments and elevational gradients [22]. Moreover, the representativeness of individual studies is questionable. For example, the highest total epiphytic biomass density on record, 44 t ha^−1^ [23], widely cited in the literature, is based on extrapolations from one tree representing each of three vegetation strata.

There is evidence to suggest that competition does not strongly shape vascular epiphyte assemblages [24,25,26], and competition may hence be less critical than in terrestrial plants. In all but the most marginal of circumstances, the species composition of terrestrial plant communities is defined by fierce competition for resources. In humid environments, these are primarily light and nutrients [27]. In subhumid to arid environments, water availability plays an increasingly important role [28]. We argue that vascular epiphytes as a life-form are fundamentally distinct in this regard. Light is plentiful in tree crowns in most environments. Although water availability is critical, it is essentially not competed for among epiphytes due to their lack of access to soil water. Any amount not intercepted and adsorbed or stored by epiphytes—the bulk of a good rainfall event—flows and drips to the ground, becoming unavailable for epiphytes. Thus, water is an ephemeral and limiting resource, albeit not strongly competed for, even in humid environments. Quite correspondingly, nutrients are yielded from atmospheric deposition and decomposing litter, absorbed from rainfall and stemflow rather than from the soil, and hence—like water—not strongly competed for [29,30]. As a consequence of these limitations, almost nowhere is there a scarcity of host bark suitable for colonisation, which is further ensured by the challenges of colonising naked bark as well as by host growth and patch disturbance (e.g., dislodgement of epiphyte mats) [25]. Rather than constituting competition, the presence of epiphytes creates niches and microsites for additional species, facilitating successful colonisation by more mesic species and hence higher species density and richness.

Numerous studies have addressed succession in epiphyte assemblages along the twig–branch trajectory, which presents neat chronosequences in any host tree, e.g., [10,31,32,33]. A characteristic of the successional process is a high degree of facilitation. Xerophilous lichens tend to pioneer the colonisation of exposed twigs, retaining water for and spores of the first bryophytes, which subsequently overgrow, gradually expand, thicken, and diversify to form increasingly moisture-retaining substrates that facilitate the establishment of many vascular species [33,34]. However, it is also vascular species themselves that facilitate other vascular epiphytes. Tank bromeliads largely owe their remarkable success to their ability to store substantial quantities of rainfall over prolonged periods. The spongy base of bromeliad tanks creates suitable microhabitats for more mesic taxa such as orchids or ferns, offering both shade and water, which continues to leak from the tank-forming leaf bases long after precipitation events. Another prime example of facilitating epiphytes is nest ferns, which store water in accumulated litter and litter-derived humus rather than tanks [35]. Countless other vascular epiphytes offer microsites to more ombrophilous taxa in a less sophisticated fashion, by providing shade and increasing rainfall and litter retention through their roots’ structuring of smooth host bark and the formation, retention, and accumulation of humus [29,36].

The apparent low competition among vascular epiphytes makes it intriguing to compare their biodiversity patterns to those of terrestrial plant life-forms. The change in species composition and richness along environmental gradients or under climate change tends to be pronounced. Such compositional change or beta diversity results from either one or, more commonly, the combination of two additive mechanisms: (a) replacement, typically by more competitive species, and (b) nestedness, where species drop out without being replaced [37]. These two mechanisms and the processes underlying them are antithetical. In order to understand how and why species assemblages change, we must discern these mechanisms and quantify their contributions to compositional change [38]. Such understanding is also a critical basis for conservation planning [39]. 

Our study site is characterised by a strong increase in humidity with elevation. We therefore expected pronounced differences in the composition and structure of plant communities. We hypothesised that (1) epiphytes compared to trees will increase more strongly in biomass (1a) and species density (1b) with elevation, due to their high sensitivity to humidity.

We further hypothesised that (2) epiphytes, being less strongly driven by resource competition, will display a greater degree of nestedness in their floristic variability among sampling plots than trees, which compete fiercely for resources. 

## 2. Results

### 2.1. Environment

Soil properties related to nutrient availability, such as pH value, C/N ratio, or plant-available phosphorus (P_resin_), tended to deteriorate slightly towards higher elevations (Table 1). Tree surface cover of both epiphytic lichens and bryophytes increased strongly at higher elevations (Table 1).

### 2.2. Biomass

**Figure 1:**
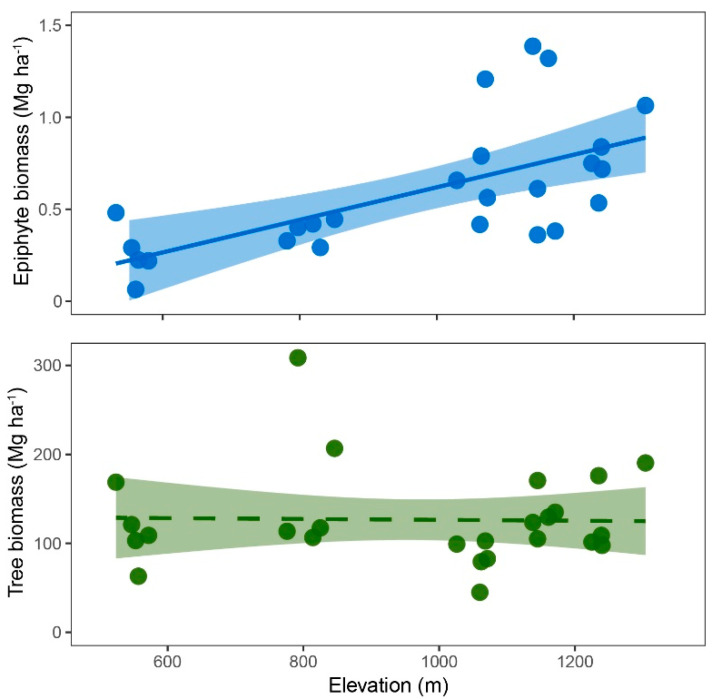
Aboveground biomass of vascular epiphytes (**top**) and trees (**bottom**) along the elevational gradient. Inserted are linear trendlines with 95% confidence intervals.

Aboveground biomass (AGB) of trees did not change significantly with elevation. Tree AGB was not correlated with elevation nor with measures of soil fertility (C/N, P, and pH; Table S1). Vascular epiphyte biomass, in contrast, increased strongly above 800 m (Figure 1), mirroring the increase in the surface cover of non-vascular epiphytes (lichens and bryophytes) (Table 1). This increase in biomass was essentially a consequence of increasing epiphyte abundance (number of stands) since the mean stand biomass varied little across elevational belts (Table 2).

### 2.3. Floristics

In total, we recorded 1128 stems from 60 tree species and 11,518 vascular epiphyte stands from 64 species.

Most speciose tree families were Fabaceae (12 species), Capparaceae (3), Moraceae (3), Polygonaceae (3), and Sapindaceae (3). The most abundant tree species were *Handroanthus chrysanthus* (Bignoniaceae, 74 stems), *Eriotheca ruizii* (Malvaceae, 68), *Ipomoea wolcottiana* subsp. *calodendron* (Convolvulaceae, 58), and *Erythrina velutina* (Fabaceae, 55). All four species are deciduous (Table S2).

Most speciose families of epiphytes were Orchidaceae (27 species) and Bromeliaceae (18), followed by Polypodiaceae (4). Across elevations, the most abundant epiphyte species were bromeliads: *Guzmania monostachia* (1600 individuals), *Tillandsia trichoglochinoides* (1170), *Vriesea spinosa* (1023), and *Tillandsia flagellata* (1018). Epiphyte assemblages at 550–800 m were composed almost entirely of Bromeliaceae, accompanied by few Cactaceae and Orchidaceae, and a stray individual of *Ficus*, whereas other angiosperm and fern taxa were only present at higher elevations (Table S3).

Our study yielded several noteworthy species records. Four epiphyte species are likely new to science (“*sp. nov.*” in Table S3). One tree species has only recently been described scientifically and is endemic to Loja province (*Pradosia aureae* [40]), while another tree species (*Schaefferia serrata*) was formerly only known from Peru.

### 2.4. Species Density and Richness

**Figure 2:**
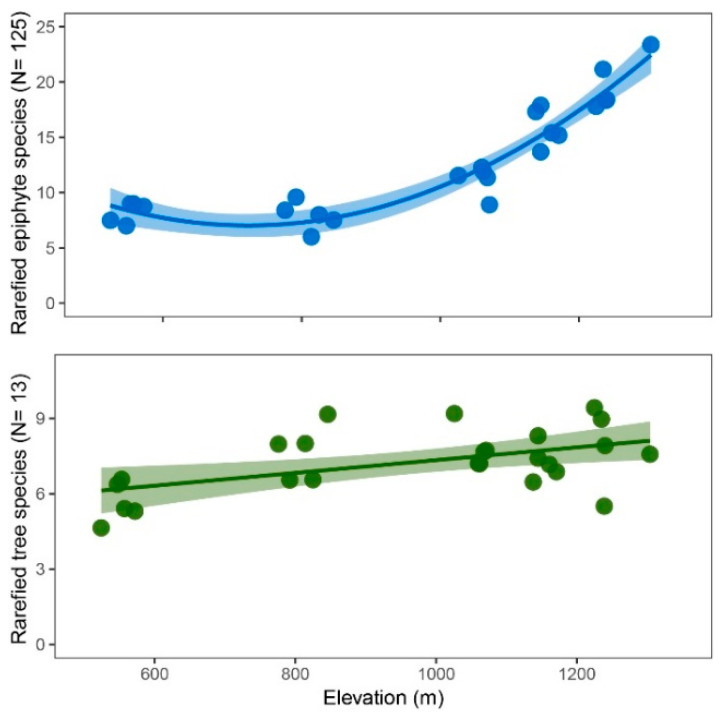
Species density (species per plot) of epiphytes (**top**) and trees (**bottom**) vs. elevation. Species numbers are calculated through individual-based rarefaction at *n* = 13 (trees) and *n* = 125 (epiphytes), respectively. Inserted are second-degree polynomial (epiphytes) and linear (trees) trendlines with 95% confidence intervals. Raw species densities are plotted in Supplementary Figure S1.

Species density was more strongly correlated with elevation in epiphytes (Spearman’s ρ = 0.88, *p* < 0.001) than in trees (ρ = 0.73, *p* < 0.001) (Table S1). While tree species density and total species richness showed a moderate linear increase with elevation, epiphyte species density and richness showed a strong increase above 800 m (Table 2). In both trees and epiphytes, this pattern persisted after controlling for variation in abundance (Figure 2).

**Figure 3:**
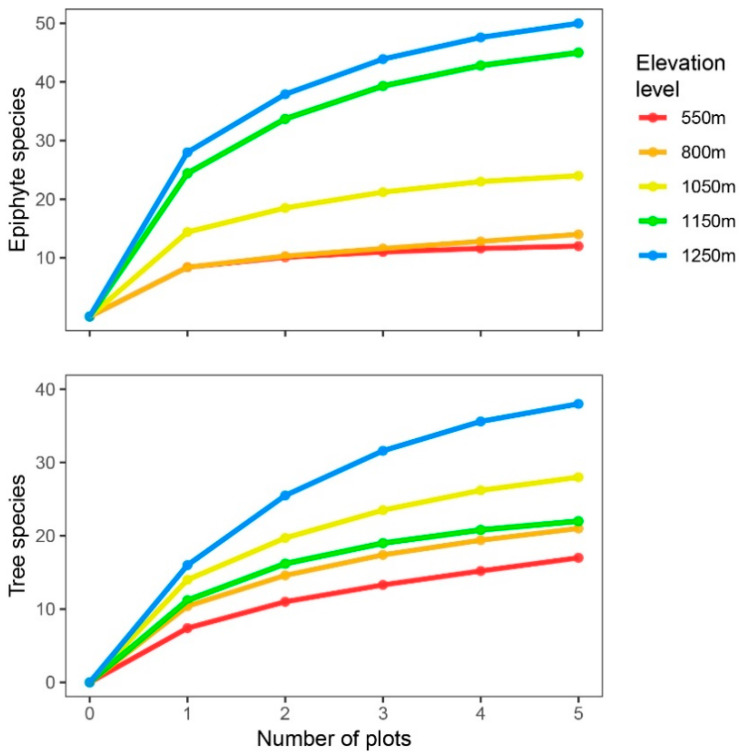
Species accumulation curves across elevational levels (strata) for epiphytes (**top**) and trees (**bottom** panel) as yielded via sample-based rarefaction (5000 runs).

The early levelling-off of species accumulation curves (Figure 3) suggests that the size and number of plots sampled were adequate to address our site’s species richness, as also confirmed by species richness estimation (Table 2).

### 2.5. Interactions between Trees and Epiphytes

Epiphyte stand number, biomass, and raw and rarefied species density showed significant (*p* < 0.01) and strong correlations (Spearman’s ρ ≥ 0.69) with elevation, lichen, and bryophyte cover. All of these vascular epiphyte measures also showed significant correlations with tree individual number (ρ = 0.63–0.67) and raw tree species density (ρ = 0.53–0.68) but not with rarefied tree species density (ρ = 0.63–0.67). No vascular epiphyte measures showed any significant correlation with tree basal area or AGB (Table S1).

### 2.6. Composition and Turnover

**Figure 4:**
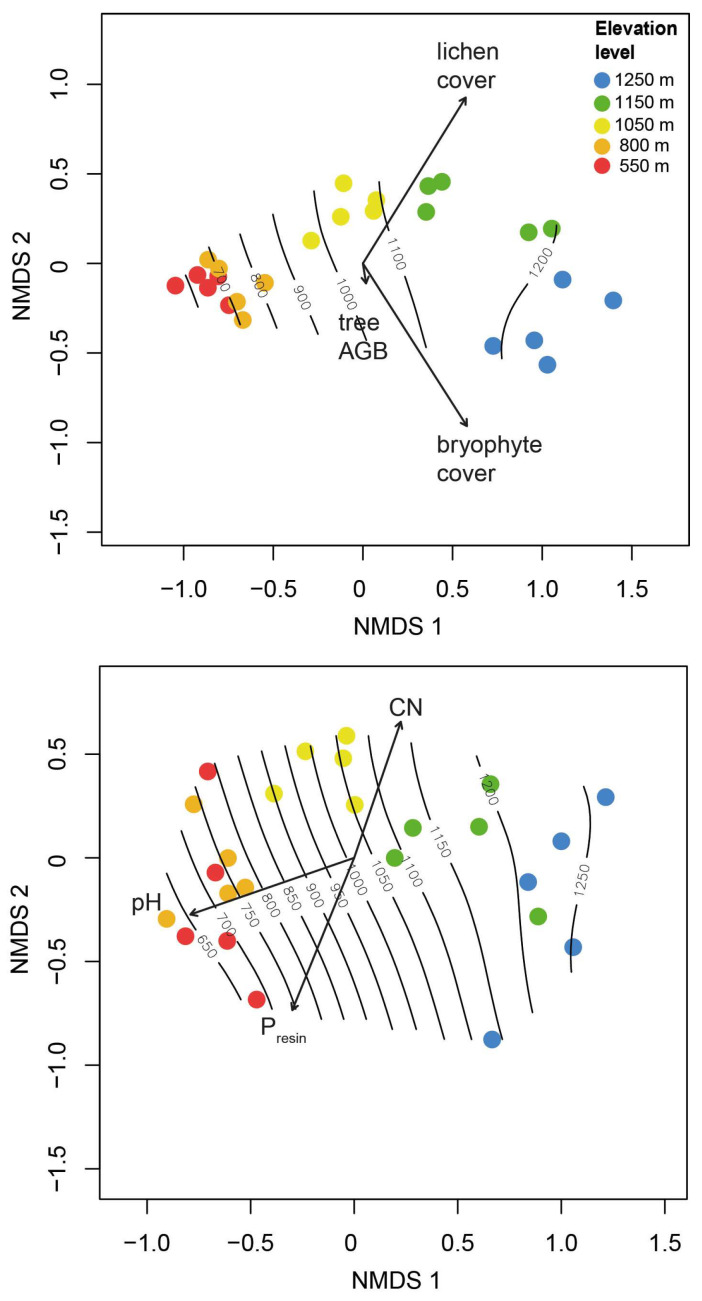
NMDS ordinations of epiphyte (**top**) and tree assemblages (**bottom**), with fitted environmental vectors and regression surfaces. The length and direction of an arrow indicate the strength and sign of the linear correlation of an environmental variable with ordination scores. The contour lines show smooth trends in the relationship between elevation and plot scores. Vectors for epiphytes: tree AGB (not slope-corrected), mean stem lichen cover, and mean bryophyte cover (covers were taken as the average between understorey and canopy values); trees: soil pH, soil C/N, and soil P_resin_.

Floristic composition was aligned closely with elevation for both epiphytes and trees (Figure 4).

**Figure 5:**
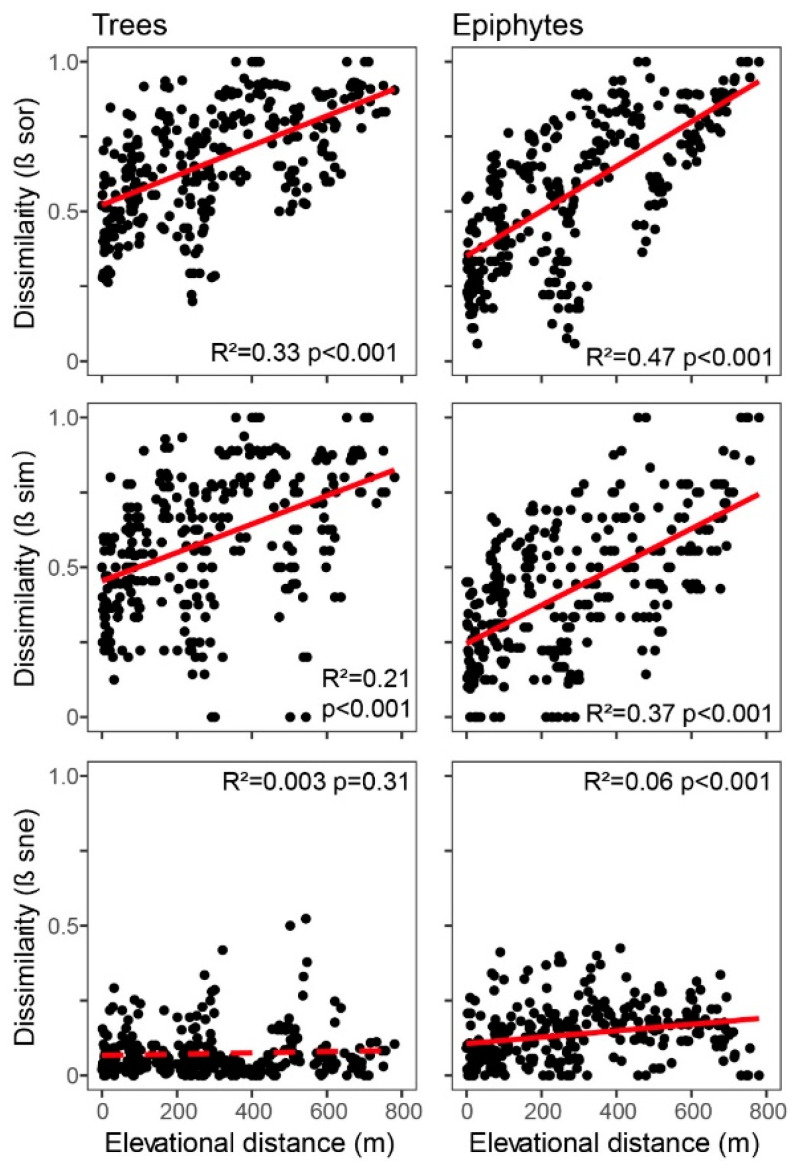
Relationship between compositional dissimilarity and plot elevational distance among trees (**left** panels) and epiphytes (**right** panels). Shown are Sørensen dissimilarity (β_sor_; **top**) and its components turnover (β_sim_; **centre**) and nestedness (β_sne_; **bottom** panels). Linear regression lines with their corresponding R^2^ and *p*-values are inserted in the panels. Differences between trees and epiphytes are significant for all three indices (paired *t*-test, df = 299): *t* = −12.2, *p* < 0.001 (β_sor_); *t* = −14.8, *p* < 0.001 (β_sim_); *t* = 8.5, *p* < 0.001(β_sne_).

Trees exhibited significantly higher levels of compositional change (Sørensen dissimilarity, β_sor_) at plot level relative to epiphytes (paired *t*-test *t* = −12.2, *p* < 0.001), and the same applied for its species turnover component (βsim; *t* = −14.8, *p* < 0.001). In contrast, the nestedness component (β_sne_) was significantly higher in epiphytes (*t* = 8.5, *p* < 0.001). All three indices (β_sor_, β_sim_, and β_sne_) increased more strongly with elevational distance in epiphytes than in trees (Figure 5).

**Figure 6:**
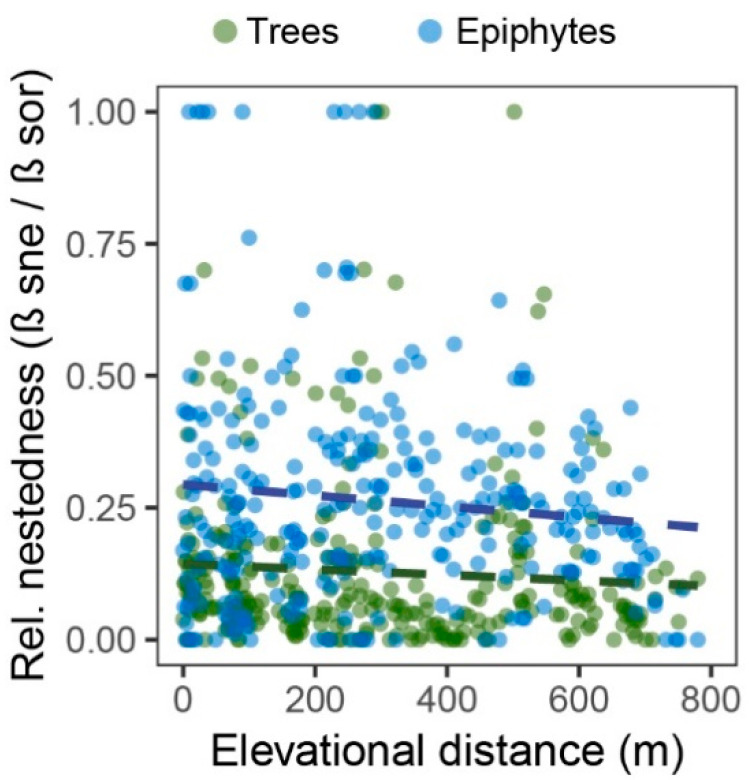
Relative nestedness (β_sne_/β_sor_) among trees (green circles) and epiphytes (blue circles). Dashed lines are linear trend lines. Relative nestedness is significantly higher in epiphytes than in trees (paired *t*-test: *t* = 9.0, df = 299, *p* < 0.001).

Epiphyte assemblages were also significantly more strongly nested in terms of relative nestedness (β_sne_/β_sor_; paired *t*-test *t* = 9.0, *p* < 0.001) (Figure 6).

## 3. Discussion

### 3.1. Biomass

Variability in AGB was fairly high, reflecting the rather small plot size for tree biomass assessments. Mean tree AGB did not exhibit a change with elevation despite variability in soil nutrient availability (Table 1 and Table 2). In general, dry forest soils are relatively fertile [28,41]. Compared to soils from Ecuadorian moist or wet forests at similar elevations [12,42], the soil nutrient availability in the studied transect is moderate to high and seems to be less limiting for plant growth than water availability. Epiphyte biomass, in contrast, increased drastically, averaging ca. threefold at 1050 m and above, relative to biomass levels at 550–800 m (Table 2). Epiphytic biomass was correlated strongly with elevation (Spearman’s ρ = 0.69; *p* < 0.01), supporting hypothesis 1a (stronger increase in epiphyte biomass with elevation). This elevational increase was paralleled by even more pronounced increases in lichen and bryophyte cover (Table 1).

Elevation does not affect biodiversity directly, but rather via its effects on climate [43]. VPD decreases from lowland towards the mid-elevation of tropical mountains and beyond, driving elevational changes in epiphyte assemblages [44,45,46,47,48]. Bryophytes are highly sensitive to humidity and VPD, and epiphytic bryophyte cover has been shown to be a good proxy for mean air humidity [49]. It is thus likely that high vascular epiphyte biomass above 800 m is likewise a result of higher levels of humidity.

In humid environments, lichen cover can decrease with increasing humidity [50]. This is partly due to increasing competition from bryophytes, especially in low-exposure microsites such as the lower canopy or understorey, e.g., [51]. Under arid conditions, in contrast, there is little evidence for such competition (see, e.g., [52,53]). Due to the markedly xeric conditions at our study site, bryophyte competition with lichens is likely to be relatively weak even at the highest elevations. Hence, the observed elevational increase in lichen cover may also result from increasing moisture availability.

### 3.2. Diversity and Composition

Species density and richness of both trees and epiphytes increased markedly with elevation. This increase was more pronounced in epiphytes, supporting hypothesis 1b (stronger increase in epiphyte species density with elevation). With respect to trees, this pattern is rather uncommon. In moist forests, the measures of tree diversity usually decline with elevation, e.g., [13,54]. Interestingly, a recent study from subhumid to humid forests in Mexico [14] found a corresponding increase in species density with elevation. We attribute this unusual pattern to a substantial decrease in hygric stress with elevation, which favours the persistence of mesic species, of which a large species pool exists in the region. Floristic composition was also aligned closely with elevation for both epiphytes and trees (Figure 4). Lichen and bryophyte covers showed a strong relationship with the floristic composition of vascular epiphytes. In contrast, this was not the case for the tree basal area or AGB.

The more drastic increase in epiphyte species richness with elevation appears to confirm their high sensitivity to VPD and drought [15,29,55]. At lower elevations, atmospheric bromeliads strongly dominated epiphyte assemblages, with more mesic taxa (e.g., aroids, ferns, Piperaceae, and most orchids) successively showing presence with increasing elevation. Unlike the lower elevations of our transect, higher elevations are subject to frequent fog, as also evidenced by an abundance of pendant moss near ridge crests (F.A.W. and J.H., pers. obs.; compare also [56,57]). Many epiphytes possess highly evolved structures (e.g., absorbing trichomes in bromeliads), which allow them to absorb condensed fog water. Therefore, water availability may effectively rise more strongly with elevation for epiphytes than for trees, which may go a long way in explaining the more dramatic increase in epiphyte species density and richness with elevation.

All epiphyte response metrics—stand number, biomass, raw and rarefied species density—showed close correlations with both lichen and bryophyte cover, suggesting a strong deterministic role of moisture availability. In contrast, none of the epiphyte response metrics showed even weak correlations with tree basal area or AGB—proxies of epiphyte substrate availability which, moreover, themselves tend to be well-correlated with stand maturity, age, and hence time for epiphyte establishment and accumulation. This lack of correlation is somewhat surprising. Typically, epiphytic biomass and species number are strongly tied to host tree size [22,58,59] and would thus be expected to correlate with tree basal area and biomass at plot level. Possibly, this causality was simply dwarfed by the strength of climate effects.

A mid-elevation peak in species numbers can be assumed to occur wherever diversity does not decline monotonously with elevation in field studies (e.g., [46,55,60,61]; but see also [62]) and database analyses [63,64]. However, in our study, species density and richness increased unabatedly, with no sign of levelling off towards the upper limit of our study gradient. Most likely, our gradient’s upper limit was too low to approach peak diversity. In the tropical Andes, vascular epiphyte species richness commonly peaks around 2000 m [64], whereas tree species richness (e.g., [13,54,65]) and tree AGB (e.g., [12]) usually decrease with elevation.

Discussing the differences in diversity patterns of trees vs. epiphytes is challenging due to a lack of references for comparison. Several studies have addressed the diversity of both trees and vascular epiphytes at plot level (e.g., [66,67,68,69,70]). However, to our knowledge, only the authors of [14] have studied both trees and epiphytes in forest plots along an elevational gradient. Their gradient of study was situated in (semi-)humid tropical E-Mexico, stretching from 0 to 3500 m a.s.l. For old-growth forests, they found that the tree species density in plots of 400 m^2^ increased slightly from sea level before peaking at 1000–1500 m. Vascular epiphytes, in comparison, showed a much more pronounced peak at 1500 m.

### 3.3. Turnover and Nestedness

We found turnover to exceed nestedness greatly in both trees and epiphytes (Figure 5). This is in line with the bulk of data sets analysed for beta diversity components, with turnover within plant assemblages typically faring over 5 x larger than nestedness [71]. However, both absolute and relative nestedness were significantly greater in epiphytes than in trees, supporting hypothesis 2 (greater nestedness in epiphytes). We attribute higher nestedness in epiphytes primarily to two factors: (a) lower resource competition among epiphytes (most notably for light, nutrients, and water) relative to trees and other terrestrial growth habits, coupled with (b) pronouncedly xeric conditions for epiphytes at our study site, particularly so at lower elevations. Patterns in epiphytic lichen and bryophyte cover at our site underpin the relevance of factor (a): tree surface cover of lichens and bryophytes increased significantly with elevation and was correlated positively with each other and with vascular epiphyte abundance and biomass (Table 1).

Strong nestedness is typical of marginal (extreme) environments. The evidence for the deterministic role of marginal growth conditions (factor b) is provided by the consistent trend in increasing nestedness with latitude towards the poles, which has been found across a variety of taxa [71]. Ref. [72] showed that land snail assemblages are nested along a gradient of habitat quality (especially soil pH). Some evidence for nestedness in marginal environments can even be found in the epiphyte literature. For a Costa Rican wet forest, [58] demonstrated how vascular epiphyte assemblages on saplings and mid-sized trees constitute nested subsets of assemblages on large trees with their more mature and diverse substrates (see also [73]). Ref. [74] found high levels of nestedness in epiphyte assemblages at the highest, frost-prone elevations (3000–3500 m a.s.l.) in tropical Mexico. In a perarid inter-Andean landscape (12 arid months), [18] found epiphyte assemblages of disturbed habitats (especially edge-exposed and isolated trees) strongly nested within the closed woodland assemblage. In this extremely xeric environment, a considerable fraction of bryophyte and vascular local flora was unable to colonise these open-canopy, highly xeric habitats, despite the close proximity of populations in closed woodlands. These species dropped out entirely, without being replaced by even more xerotolerant species.

Floristic similarity for both epiphytes and trees decreased as elevational distance between plots rose, as would be expected as a consequence of gradually diverging environmental conditions (e.g., [75]). Absolute nestedness increased with increasing elevational distance between pairs of plots. This pattern may reflect communities at low elevations being subsets of high-elevation communities to a degree, with the resulting increase in nestedness being outpaced by species turnover as would be expected along such a lengthy gradient in a landscape with a large species pool. We attribute this result to (a) lower resource competition among epiphytes (most notably for light, nutrients, and water) relative to trees and other terrestrial growth habits, coupled with (b) extremely xeric conditions for epiphytes at our study site, especially at lower elevations.

The relationship between absolute pairwise nestedness and elevational distance between plots may not be linear but rather hump-shaped, with nestedness returning to shrink with increasing elevational distance beyond a certain gradient length. This seems reasonable to expect because few species are sufficiently plastic to inhabit both extremes of very long gradients. In fact, somewhat lower levels of nestedness at our largest elevational distances (i.e., 700–800 m, Figure 5) may possibly indicate that a respective inflection point was already passed in our gradient of study.

## 4. Methods

### 4.1. Study Site

Field work was carried out from March to September 2010 in the private protected area *Reserva Natural Laipuna*, Loja Province, southwestern Ecuador. The reserve is situated on the northern rim of the Andes’ Huancabamba depression, where the exceptionally low stature of the Andes permits a peculiar mosaic of humidity conditions and facilitates species exchange between eastern and western Andean slopes [76,77]. The reserve constitutes one of the few well-preserved elevational gradients of Tumbesian dry forest [78,79]. Tumbesian dry forests span a narrow strip of approximately 50,000 km^2^ [80] located between the Pacific Ocean and the Andes, extending from the southwestern tip of Ecuador to northwestern Peru. These forests are found at elevations ranging from sea level to mostly below 1500 m a.s.l. The rainy season lasts from January to May, and the dry season lasts from June to December. At 600 m a.s.l., annual mean temperature is 23.7 °C and annual rainfall is approximately 540 mm, with high year-on-year variability [81,82]. Higher altitudes receive additional moisture input, partly from fog driven by westerly winds [83]. At 1450 m a.s.l., annual mean temperature is 16.1 °C and rainfall is approximately 1260 mm [84]. The forest is deciduous, with an increasing number and abundance of semi-deciduous and evergreen species at higher elevation. Canopy height averages 11–16 m regardless of elevation, with tallest tree individuals reaching up to 20 m. The reserve has been under protection since 2002. Selective logging and extensive goat and cattle foraging had affected the forest in earlier decades [79].

### 4.2. Forest Inventory

**Figure 7:**
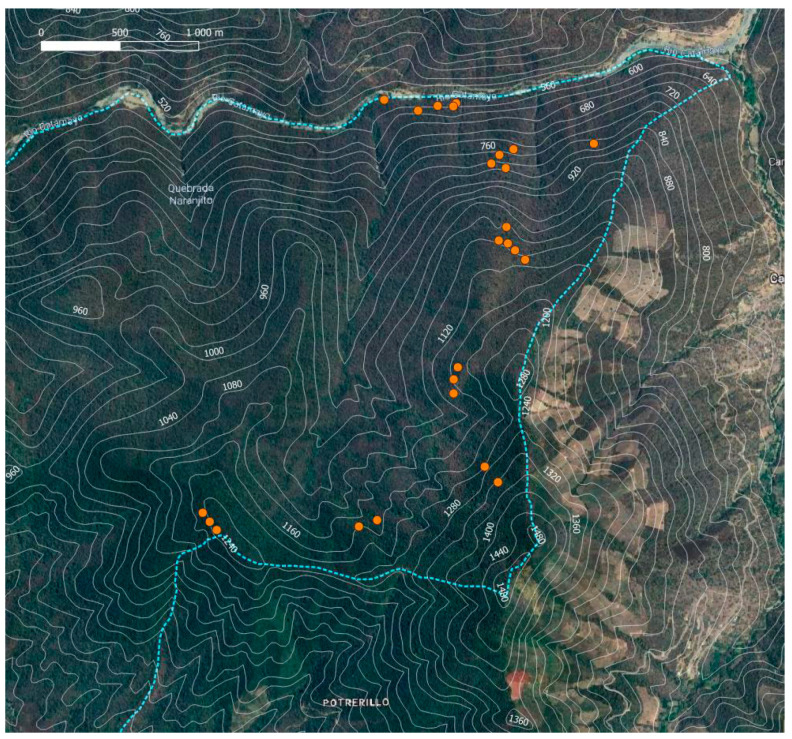
Map of the study area Reserva Natural Laipuna (reserve border: stippled blue line) and plot locations (orange dots).

We stratified the ca. 800 m of elevational range accessible within the reserve into five elevational levels. Within each elevational stratum, we laid out five plots of 20 × 20 m (in total 25 plots, at 525–1304 m a.s.l.) (Figure 7). For simplicity, these elevational strata are referred to as 550, 800, 1050, 1150, and 1250 m a.s.l. in this paper. Plots were positioned randomly within mature sections of the reserve, avoiding major recent disturbances such as landslides or larger tree fall gaps.

All trees ≥5 cm dbh (diameter at breast height, 130 cm) were recorded and tagged, measuring tree height with a Vertex IV height meter and a T3 transponder (Haglöf, Langsele, Sweden).

Facultative and obligatory vascular holo-epiphytes and primary hemi-epiphytes (*sensu* [85]) were recorded for all woody host plants rooted within the plots. We counted the number of stands (an individual or indiscernibly dense groups of individuals of one species; [86]) rooted at >0.25 m above ground using binoculars while omitting seedlings and early juveniles. We modified the method of [60] for assessing epiphytic biomass: for each species and plot, we collected one specimen representative of a mean-sized stand using a telescope pruning pole. If such a stand could not be collected inside a plot, it was sought in the surroundings. In rare cases, it was approximated by dividing a larger stand to the right size or by assigning an estimated correction factor to a smaller stand. Primary hemi-epiphytes (*Ficus, Clusia*) with soil contact were excluded from biomass sampling and analysis.

Species identifications were made and specimens were deposited at the herbaria LOJA, QCA, and QCNE and at the institutional herbaria of selected taxonomic experts (see Acknowledgments).

The percentage of bryophyte and lichen cover of living woody substrate surfaces was estimated visually for each plot quadrant for understorey (conservatively defined as 0.25–2.25 m above ground) and canopy (>2.25 m), respectively. Vascular epiphyte cover was not recorded but probably did not exceed ca. 10% of woody plant surface in any of the plots (F.A.W., pers. obs.).

A topsoil sample representative of 0–10 cm depth was taken in the centre of each plot quadrant, lumped into one mixed sample per plot, and air-dried for lab analysis at the Department of Plant Ecology, University of Göttingen, Germany, following the analytical methods of [42].

### 4.3. Epiphyte and Tree Biomass Estimation

Epiphyte biomass samples were stored in paper bags and air-dried to constant weight in the lab at ca. 40 °C before determining dry weight on a digital scale. Biomass was then calculated for each plot by multiplying individual numbers by the dry weight of the sampled specimen. We consider this method to be very suitable for our study site characterised by rather low diversity coupled with regular occurrence of most epiphyte species.

We used the allometric equation developed by [87] specifically for tropical dry forests to estimate tree aboveground biomass (AGB):

AGB = 0.112 × (ρD^2^ H)^0.916^

where ρ is wood specific gravity (oven-dried wood mass over green volume), D is trunk dbh, and H is tree height in metres. Wood density data were taken from [88,89].

Since plots were laid out on 400 m^2^ of land surface area we considered plot inclination for calculating biomass ha^−1^ and corrected the actual plot area by dividing plot length by cos α (with α being the inclination angle).

### 4.4. Data Analysis

Because plant abundance varied greatly among plots, we applied individual-based rarefaction to remove the effects of abundance on species density (species per plot) using PAST 4.03 [90]. Sample-based rarefaction and species richness estimations (bias-corrected Chao, Jackknife, and Michaelis–Menten mean) were calculated with EstimateS 8.2 [91]. Community composition was analysed using non-metric multidimensional scaling using Vegan 2.6-4 in R [92].

We followed [37] in separating beta diversity (Sørensen dissimilarity; β_sor_) into its additive components, species turnover and nestedness-resultant turnover (termed nestedness henceforth). In contrast to other metrics for analysing beta diversity, the turnover component of the Baselga family of metrics is independent of differences in species richness [93]. We calculated Sørensen dissimilarity (β_sor_) and its components species turnover (β_sim_) and nestedness (β_sne_) using betapart 1.6 in R [94] and compared the respective results for epiphytes and trees with paired *t*-tests.

Statistics were carried out using R 4.3.3 [95] unless stated otherwise.

## 5. Conclusions

Both trees and vascular epiphytes displayed increasing species density and richness with increasing elevation. Those of epiphytes, however, increased more strongly. Unlike trees, epiphytes also increased markedly in their projected biomass and as a function of increasing abundance. The plot-level compositional variability was similarly strong for both life-forms but with significantly higher nestedness in epiphytes than in trees.

We attribute the elevational increases in the species richness of trees and epiphytes to increasing humidity, i.e., more mesic growth conditions at higher elevations. The more pronounced elevational increase in epiphyte biomass, species density, and richness—the latter coupled with a higher degree of nestedness—we attribute to greater moisture dependency of epiphytes and low direct competition for resources (light, water, nutrients) among epiphytes.

To our knowledge, this study is novel in comparing epiphytes to terrestrial plants with regard to beta diversity components, i.e., the mechanisms of nestedness vs. species turnover, revealing a markedly distinct pattern for epiphytes. The notion that, unlike terrestrial plant life-forms, vascular epiphyte assemblages may not be driven by intense competition has long been implicit in epiphyte ecology. However, to our knowledge, it has not been tested in general terms, nor have its potential ecological implications received due attention (but see, e.g., [30,73]).

In ecosystems such as cloud forests, where growth conditions are ideal and plant density is very high, competition among vascular epiphytes may well be substantial [26]. In general, however, across life zones and with sparse exceptions, the vascular epiphyte cover on woody host surfaces is low [25], by and large remaining well below ca. 20–25% throughout the tropics (F.A.W., pers. obs.). This holds true even for many of the most speciose ecosystems, suggesting that comparably low competition among vascular epiphytes may be a phenomenon of general validity. If true, this circumstance has wide implications for epiphyte diversity and its underlying processes. For instance, low competitive pressure could help explain the volatility of epiphyte assemblages in terms of species density, as well as the remarkably high epiphyte α and γ diversity of some forest landscapes (e.g., [15,96,97]). It could further suggest that vascular epiphyte communities are more strongly driven by stochastic processes than the terrestrial life-forms of plants. Future studies will need to clarify if and how low levels of competition affect the diversity and abundance of vascular epiphytes. 

To our knowledge, our study further provides a first comparison of elevational patterns in epiphyte and tree diversity and biomass for a tropical dry forest. It revealed considerable species richness and yielded several new species registers, underlining the poor status of floristic knowledge on and high relevance of tropical montane dry forests for global biodiversity conservation. Our results further highlight the importance of preserving contiguous elevational gradients for biodiversity conservation in the face of global climate change.

## Figures and Tables

**Table 1:** Environmental characteristics (mean ± standard deviation for every 5 plots per elevational stratum).

	550 m			800 m			1050 m			1150 m			1250 m		
	Mean	±	SD	Mean	±	SD	Mean	±	SD	Mean	±	SD	Mean	±	SD
Elevation (m a.s.l.)	550.6	±	17.5	810.6	±	27.5	1057.4	±	18.1	1152.0	±	13.6	1248.6	±	31.5
Slope inclination (°)	30.6	±	4.8	29.8	±	8.1	22.0	±	3.8	26.5	±	6.3	29.2	±	4.2
Soil pH (KCl)	6.5	±	0.1	6.6	±	0.1	5.9	±	0.1	5.7	±	0.2	5.9	±	0.3
Soil N_total_ (%)	0.2	±	0.0	0.3	±	0.0	0.3	±	0.1	0.3	±	0.1	0.6	±	0.1
Soil C/N	10.1	±	1.0	10.7	±	0.7	11.6	±	0.5	11.2	±	0.5	11.0	±	0.3
Soil P_resin_ (µmol g^−1^)	0.6	±	1.2	2.5	±	0.9	0.5	±	0.2	1.4	±	0.7	1.9	±	1.0
Soil cation exchange capacity (µmol g^−1^)	23.1	±	29.6	58.0	±	42.1	32.4	±	22.6	176.1	±	202.5	296.5	±	206.3
Soil base saturation (%)	99.7	±	0.3	95.0	±	11.3	89.0	±	22.5	88.6	±	11.5	88.8	±	6.8
Lichen cover understorey (%)	1.2	±	0.4	7.0	±	3.7	26.6	±	15.7	37.8	±	12.4	24.0	±	9.5
Lichen cover canopy (%)	2.2	±	2.2	3.0	±	2.7	29.4	±	11.5	46.4	±	7.8	33.6	±	13.3
Bryophyte cover understorey (%)	1.2	±	0.4	1.0	±	0.0	7.4	±	5.9	9.6	±	2.7	41.4	±	6.8
Bryophyte cover canopy (%)	1.0	±	0.0	1.0	±	0.0	3.6	±	2.9	4.8	±	1.5	18.6	±	4.0

**Table 2:** Elevational trends in abundance, biomass, and diversity of trees and vascular epiphytes (mean ± standard deviation for 5 plots per elevational stratum).

	550 m			800 m			1050 m			1150 m			1250 m		
	Mean	±	SD	Mean	±	SD	Mean	±	SD	Mean	±	SD	Mean	±	SD
**Trees**															
Individuals	22.2	±	3.8	24.4	±	8.5	43.8	±	5.1	33.0	±	8.7	48.8	±	9.1
Basal area (cm^2^ plot^−1^)	8902	±	2773	17,453	±	9400	10,695	±	1398	9838	±	2735	10,081	±	2595
AGB (Mg ha^−1^) ^a^	113	±	38	171	±	87	82	±	23	133	±	24	135	±	45
Species density raw	7.4	±	1.5	10.4	±	3.2	14.0	±	1.6	11.2	±	1.5	16.0	±	3.7
Species density rarefied (*n* = 13)	5.7	±	0.8	7.6	±	1.1	7.8	±	0.8	7.2	±	0.7	7.9	±	1.5
Species richness observed	17			21			28			22			38		
Species richness estimated															
Chao 1	20.3	±	4.1	28.0	±	7.1	29.3	±	1.7	24.5	±	3.2	39.3	±	1.6
Chao 2	31.4	±	13.1	25.5	±	4.4	30.9	±	2.8	23.7	±	2.1	41.1	±	2.7
Jackknife 1	24.2	±	1.5	27.4	±	3.5	35.2	±	1.5	26.8	±	2.3	47.6	±	2.7
Jackknife 2	29.2			30.4			36.6			27.7			47.6		
Michaelis–Menten (mean)	24.2			27.6			37.0			29.1			59.0		
**Epiphytes**															
Stands (no.)	176	±	56	253	±	25	570	±	339	700	±	178	604	±	287
Plant dry weight (g) ^b^	47.7	±	19.1	52.0	±	12.6	51.4	±	14.6	38.3	±	15.6	52.1	±	25.3
Biomass (Mg ha^−1^ slope-corrected)	0.26	±	0.15	0.38	±	0.06	0.73	±	0.30	0.81	±	0.50	0.78	±	0.19
Species density raw	8.4	±	0.9	8.4	±	1.5	14.4	±	2.9	24.4	±	4.0	27.6	±	3.1
Species density rarefied (*n* = 125)	8.2	±	0.9	7.9	±	1.3	11.2	±	1.3	15.9	±	1.7	19.8	±	2.4
Species richness observed	12			14			24			45			50		
Species richness estimated															
Chao 1	12.0	±	0.2	17.0	±	4.2	25.5	±	2.6	45.8	±	1.3	59.3	±	8.9
Chao 2	12.3	±	0.8	26.0	±	11.0	24.9	±	1.3	48.1	±	2.8	53.8	±	3.2
Jackknife 1	13.6	±	1.0	18.8	±	1.5	28.0	±	1.3	53.8	±	3.4	59.6	±	2.4
Jackknife 2	13.9			22.4			27.4			54.6			61.0		
Michaelis–Menten (mean)	13.3			15.8			28.6			57.0			62.0		

^a^ slope-corrected (see Methods section for details). ^b^ calculated as total biomass × number of stands^−1^.

## Data Availability

Epiphyte data are available through the EpiG database [98]; https://epigdatabase.weebly.com/). Tree data are available via the DFG-FOR816 Data Warehouse at http://www.tropicalmountainforest.org.

## Supplementary Materials

The following supporting information can be downloaded at https://www.mdpi.com/article/10.3390/plants13182555/s1. Table S1. Correlations among the selected response and predictor variables. Table S2. Tree individuals (≥5 cm dbh) across the 25 study plots. Table S3. Vascular epiphyte abundance (‘stands’ *sensu* Sanford 1968) across the 25 study plots. Figure S1. Raw species density (species per plot) of epiphytes (top) and trees (bottom) vs. elevation.
