# Supplementary material for: Diverging Elevational Patterns of Tree vs. Epiphyte Species Density, Beta Diversity, and Biomass in a Tropical Dry Forest
Source: Plants (Basel). 2024 Sep 11;13(18):2555. doi: 10.3390/plants13182555 (PMC11434910; doi:10.3390/plants13182555)
Supplement: Supplementary file 1 [file plants-13-02555-s001.zip › plants-3122677-supplementary.pdf]

Supplementary materials

Table S1. Correlations among selected response and predictor variables. Correlation coefficients are given as Spearman's ϱ. Correlations significant at $p < 0.01$ are shown in bolt font.

	Eleva-tion (m a.s.l.)	Soil pH	Soil N_{total}	Soil C/N	Soil P_{resin}	Lichen cover	Bryo. cover	Tree inds.	Tree basal area	Tree bio-mass	Tree spp. raw	Tree spp. raref.	Epiph. stands	Epiph. bio-mass	Epiph. spp. raw
Soil pH (KCl)	**-0,71**														
Soil N_{total} (%)	**0,69**	-0,40													
Soil C/N	0,35	-0,35	0,47												
Soil P_{resin} (µmolP g^{-1})	-0,50	**0,52**	-0,43	**-0,64**											
Lichen cover (%) [a]	**0,73**	**-0,71**	0,48	**0,56**	**-0,62**										
Byophyte cover (%) [a]	**0,90**	**-0,67**	**0,67**	0,35	-0,38	**0,71**									
Tree individuals (no.)	**0,77**	**-0,57**	**0,53**	0,39	**-0,63**	**0,58**	**0,77**								
Tree basal area (cm^2 $plot^{-1}$)	-0,11	0,20	0,10	0,18	-0,20	-0,09	-0,21	-0,02							
Tree biomass (Mg $plot^{-1}$) [b]	0,09	0,17	-0,13	-0,20	0,16	0,00	-0,03	-0,14	**0,56**						
Tree spp. density raw (no.) [c]	**0,73**	-0,49	**0,66**	0,40	**-0,62**	**0,58**	**0,62**	**0,78**	0,22	-0,02					
Tree spp. density raref. (no; n=13)	0,42	-0,20	**0,47**	0,27	**-0,41**	**0,30**	0,22	0,33	0,32	-0,01	0,79				
Epiphyte stands (no.)	**0,77**	**-0,71**	**0,51**	**0,58**	**-0,62**	**0,86**	**0,76**	**0,64**	0,10	0,12	**0,60**	0,34			
Epiphyte biomass (Mg $plot^{-1}$) [b]	**0,69**	**-0,60**	**0,52**	**0,53**	**-0,55**	**0,71**	**0,71**	**0,63**	0,05	0,01	**0,53**	0,32	**0,84**		
Epiphyte spp. density raw (no.)	**0,88**	**-0,75**	**0,64**	**0,38**	-0,45	**0,80**	**0,87**	**0,65**	-0,17	0,00	**0,59**	**0,25**	**0,80**	**0,67**	
Epiphyte spp. density raref. (no; n=125)	**0,88**	**-0,75**	**0,62**	0,31	-0,38	**0,71**	**0,86**	**0,67**	-0,21	-0,02	**0,61**	0,24	**0,73**	**0,59**	**0,96**

[a] Lichen and bryophyte covers are means calculated from understorey and canopy values.
[b] Tree and epiphyte biomass values uncorrected for slope.

Table S2. Tree individuals (≥ 5 cm dbh) across the 25 study plots.

			550 m					800 m					1050 m					1150 m					1250 m				
**Family**	**Species**	**Col. no.**[a]	**1**	**2**	**3**	**4**	**5**	**1**	**2**	**3**	**4**	**5**	**1**	**2**	**3**	**4**	**5**	**1**	**2**	**3**	**4**	**5**	**1**	**2**	**3**	**4**	**5**
Achatocarpaceae	*Achatocarpus pubescens*	1321	0	0	0	1	0	1	0	0	0	0	0	0	0	0	0	0	0	0	0	0	0	0	0	0	0
Anacardiaceae	*Loxopterygium huasango*	4293	0	0	0	0	3	0	0	3	1	0	1	0	0	1	0	0	0	0	0	0	0	0	0	0	0
Araliaceae	*Aralia excelsa*	6256	0	0	0	0	0	0	0	0	0	0	0	0	0	0	0	0	0	0	0	0	0	0	0	1	0
Asteraceae	*Dasyphyllum popayaense*	1360	0	0	0	0	0	0	0	0	0	0	0	0	0	0	0	0	0	0	6	7	1	6	1	0	1
Asteraceae	*sp.*	–	0	0	0	0	0	0	0	0	0	0	0	0	0	0	0	0	0	0	0	0	0	0	1	0	0
Bignoniaceae	*Handroanthus chrysanthus*	4282	0	0	0	0	0	0	0	0	0	0	0	2	0	0	0	11	9	4	4	7	18	2	5	3	9
Bignoniaceae	*Tecoma castanifolia*	1318	0	0	1	0	0	0	0	0	0	4	0	0	0	0	0	0	0	0	0	0	0	0	0	0	0
Bixaceae	*Cochlospermum vitifolium*	1340	1	1	1	3	0	1	0	2	0	1	0	0	0	0	0	0	0	0	0	0	0	0	0	0	0
Boraginaceae	*Cordia alliodora*	–	0	0	0	0	0	0	0	0	0	0	0	0	0	0	0	0	0	0	0	0	0	1	3	0	2
Boraginaceae	*Cordia lutea*	–	4	1	1	9	0	1	6	9	0	5	0	0	0	0	0	0	0	0	0	0	0	0	0	0	0
Burseraceae	*Bursera graveolens*	1312	0	0	3	1	5	2	1	6	3	5	1	3	0	2	2	0	0	0	0	0	0	0	0	0	0
Cannabaceae	*Celtis loxensis*	4292	0	0	0	0	0	0	0	0	0	0	1	0	1	0	2	0	0	1	0	0	0	0	0	0	0
Capparaceae	*Morisonia flexuosa*	6246	0	0	0	0	0	0	1	0	1	1	0	0	1	1	0	0	0	0	0	0	0	0	0	0	0
Capparaceae	*Morisonia petiolaris*	6147	0	0	0	0	0	0	0	0	0	0	0	0	0	0	0	0	0	0	0	1	0	6	1	0	0
Capparaceae	*Morisonia scabrida*	1315	0	3	0	0	0	0	0	0	1	0	0	1	1	0	0	0	0	0	0	0	0	0	0	0	0
Caricaceae	*Vasconcella candicans*	1328	0	0	0	0	0	0	0	0	0	0	0	0	0	0	0	0	0	0	0	0	1	0	0	0	0
Celastraceae	*Maytenus sp.*	4303	0	0	0	0	0	0	0	0	0	0	0	0	0	0	0	0	0	0	0	0	2	0	0	0	0
Celastraceae	*Salacia sp.*	6255	0	0	0	0	0	0	0	0	0	0	1	6	6	4	3	0	0	0	0	0	0	0	0	0	0
Celastraceae	*Schaefferia serrata*	6146	0	0	0	0	0	0	0	0	0	0	0	0	0	0	0	0	0	0	0	0	2	0	0	0	0
Combretaceae	*Terminalia valverdae*	4284	0	0	0	0	0	0	0	0	0	0	9	0	0	0	0	0	1	0	2	2	4	2	0	1	1
Convolvulaceae	*Ipomoea wolcottiana*	1351	0	2	5	1	7	3	2	1	4	3	3	4	8	3	3	5	0	4	0	0	0	0	0	0	0
Fabaceae	*Bauhinia ayabacensis*	6244	0	0	0	0	0	0	0	0	0	0	1	0	2	4	7	0	0	0	1	0	7	1	0	0	0
Fabaceae	*Cyathostegia mathewsii*	6250	0	0	0	0	0	0	0	0	0	0	1	0	0	4	0	0	0	0	1	0	0	0	2	0	0
Fabaceae	*Erythrina velutina*	–	0	0	2	0	1	0	0	0	4	2	1	5	5	2	10	5	7	9	2	0	0	0	0	0	0
Fabaceae	*Geoffrea spinosa*	6248	0	0	0	1	0	0	0	0	0	1	0	1	0	0	1	0	2	0	0	1	1	1	0	0	0
Fabaceae	*Leucaena trichodes*	1354	1	14	1	0	0	0	1	1	0	1	8	1	1	4	1	5	1	1	1	0	0	1	2	0	0
Fabaceae	*Libidibia glabrata*	–	2	0	0	0	0	0	0	0	0	0	0	0	0	0	0	0	0	2	0	0	0	0	0	0	0
Fabaceae	*Machaerium millei*	6460	0	0	0	0	0	0	0	0	0	0	11	14	10	0	7	0	0	0	0	0	0	0	0	0	0
Fabaceae	*Piptadenia retusa*	1305	0	1	0	0	0	0	0	0	0	0	0	0	0	0	1	0	0	0	1	0	2	0	0	0	0
Fabaceae	*Piscidia carthagenensis*	1320	0	0	0	0	0	1	0	0	10	1	0	0	0	0	0	0	0	0	0	0	0	0	0	0	0
Fabaceae	*Pseudalbizzia multiflora*	4298	0	0	0	0	0	0	1	0	0	0	0	0	0	2	1	0	0	0	0	0	0	1	1	0	0
Fabaceae	*Senna mollissima*	4297	0	0	0	0	0	0	0	0	0	0	0	0	0	1	1	0	0	0	0	0	0	0	2	0	0
Fabaceae	*Vachellia macracantha*	–	0	0	0	0	0	0	0	0	0	0	0	0	1	0	0	1	1	0	0	0	0	0	0	0	0
Lauraceae	*Ocotea sp.*	6461	0	0	0	0	0	0	0	0	0	0	0	0	0	0	0	0	0	0	0	0	0	0	0	3	1
Malvaceae	*Ceiba trichistandra*	1316	1	1	1	0	0	1	1	3	2	2	0	0	0	3	0	0	0	0	0	0	0	0	0	0	0

			550 m					800 m					1050 m					1150 m					1250 m				
**Family**	**Species**	**Col. no.**	**1**	**2**	**3**	**4**	**5**	**1**	**2**	**3**	**4**	**5**	**1**	**2**	**3**	**4**	**5**	**1**	**2**	**3**	**4**	**5**	**1**	**2**	**3**	**4**	**5**
Malvaceae	*Eriotheca ruizii*	–	8	5	8	6	2	3	3	2	4	3	4	1	1	4	4	1	0	1	1	2	3	0	0	1	1
Meliaceae	*Schmardea microphylla*	4302	0	0	0	0	0	0	0	0	0	0	0	0	0	0	0	0	0	0	0	0	0	2	0	0	3
Moraceae	*Ficus jacobii*	4489	0	0	0	0	0	0	0	0	0	0	0	0	0	0	0	0	0	0	0	0	0	0	1	0	0
Moraceae	*Ficus sp.*	6225	0	0	0	0	0	0	0	0	0	0	0	4	1	0	0	3	1	0	1	0	0	0	6	0	7
Moraceae	*Maclura tinctoria*	1348	0	0	0	0	0	0	0	0	0	0	0	0	0	0	0	0	0	0	0	0	0	0	1	1	0
Myrtaceae	*Myrcia splendens*	4488	0	0	0	0	0	0	0	0	0	0	0	0	0	0	0	0	0	0	0	0	0	1	0	0	0
Myrtaceae	*Psidium rostratum*	5274	0	0	0	0	0	0	0	0	0	0	0	0	0	0	0	1	4	0	5	1	2	0	1	0	1
Nyctaginaceae	*Bougainvillea peruviana*	1326	0	0	0	0	0	0	0	0	0	1	0	0	0	1	0	0	0	0	0	0	2	1	0	0	0
Nyctaginaceae	*Pisonia aculeata*	4279	1	0	0	0	0	0	1	1	0	0	1	0	0	1	0	2	1	1	0	0	1	1	0	1	0
Opiliaceae	*Agonandra excelsa*	6143	0	0	0	0	0	0	0	0	0	1	0	0	0	0	0	0	0	0	0	0	0	1	0	0	1
Phyllanthaceae	*Phyllanthus sp.*	5275	0	0	0	0	0	0	0	0	0	0	2	3	4	0	0	3	2	5	1	1	10	2	3	0	2
Polygonaceae	*Coccoloba ruiziana*	4246	2	0	0	0	0	0	0	0	0	0	0	0	0	0	0	0	0	0	0	0	0	0	0	0	0
Polygonaceae	*Ruprechtia aperta*	4299	0	0	0	0	0	0	0	0	0	0	0	0	0	0	0	0	0	7	2	1	0	0	1	0	3
Polygonaceae	*Triplaris cumingiana*	6144	0	0	0	0	0	0	0	0	0	0	0	0	0	0	0	0	0	0	0	0	3	5	5	5	0
Rhamnaceae	*Sarcomphalus thyrsiflora*	4285	0	0	0	0	0	0	0	0	0	1	1	0	0	0	0	0	0	0	0	0	0	0	0	0	0
Rubiaceae	*Simira ecuadorensis*	6257	0	0	0	0	0	0	0	0	0	0	5	0	0	0	0	0	0	0	0	0	0	0	0	0	0
Rutaceae	*Zanthoxylum fagara*	6145	0	0	0	0	0	0	0	0	0	0	0	0	0	0	0	0	0	0	0	0	0	0	1	0	1
Salicaceae	*Prockia pentamera*	6458	0	0	0	0	0	0	0	0	0	0	0	0	0	0	0	7	0	4	0	0	2	0	1	0	0
Salicaceae	*Xylosma sp.*	4493	0	0	0	0	0	0	0	0	0	0	0	0	0	0	0	0	0	0	0	0	0	0	0	0	1
Sapindaceae	*Allophylus cf. mollis*	6459	0	0	0	0	0	0	0	0	0	0	0	0	0	0	0	0	0	0	0	0	0	0	1	7	7
Sapindaceae	*Cupania sp.*	4491	0	0	0	0	0	0	0	0	0	0	0	0	0	0	0	0	0	0	0	0	0	0	0	0	2
Sapindaceae	*Sapindus saponaria*	1334	0	0	0	0	0	0	0	0	0	0	0	0	0	0	1	0	1	1	0	0	0	0	0	0	0
Sapotaceae	*Pradosia aureae*	6252	0	0	0	0	0	0	0	0	0	0	0	0	0	0	0	0	0	0	0	0	0	0	0	22	12
Solanaceae	*Acnistus arborescens*	4936	0	0	0	0	0	0	0	0	0	0	0	0	0	0	0	0	0	0	0	0	0	4	6	0	0
Solanaceae	*Solanum sp.*	4295	0	0	0	0	0	0	1	0	0	1	0	0	0	0	0	0	0	0	0	0	0	0	0	0	0

[a] Specimen collection numbers (col. no.) refer to collector Jürgen Homeier.

Table S3. Vascular epiphyte abundance ('stands' *sensu* Sanford 1968) across the 25 study plots.

		Col.	550 m					800 m					1050 m					1150 m					1250 m				
**Family**	**Species**	**ref.**[a]	**1**	**2**	**3**	**4**	**5**	**1**	**2**	**3**	**4**	**5**	**1**	**2**	**3**	**4**	**5**	**1**	**2**	**3**	**4**	**5**	**1**	**2**	**3**	**4**	**5**
Araceae	*Anthurium boekei*	915	0	0	0	0	0	0	0	0	0	0	0	0	0	0	0	0	0	0	6	5	51	34	17	31	39
Araceae	*Anthurium dombeyanum*	2982	0	0	0	0	0	0	0	0	0	0	0	0	0	0	0	0	0	0	5	29	24	22	13	35	11
Araceae	*Anthurium pohlianum*	953	0	0	0	0	0	0	0	2	0	0	1	2	0	0	0	32	15	34	29	1	10	10	0	0	1
Bromeliaceae	*Guzmania monostachia*	921	0	0	0	0	0	0	0	5	0	0	174	65	29	6	36	211	85	185	178	241	17	16	127	74	151
Bromeliaceae	*Pitcairnia prolifera*	904	0	0	0	0	0	0	1	0	0	0	0	1	0	0	0	0	0	0	24	32	114	55	10	4	2
Bromeliaceae	*Racinaea multiflora*	925	22	14	8	20	22	92	22	19	42	21	90	80	87	59	26	74	24	27	17	77	0	5	6	2	4
Bromeliaceae	*Racinaea sp.*	1646	0	0	0	0	0	0	0	0	0	0	0	0	0	0	0	0	0	0	0	0	1	0	0	0	0
Bromeliaceae	*Racinaea pugiformis*	926	0	0	0	0	0	0	0	0	0	0	0	0	0	0	0	123	66	21	11	11	54	13	106	3	162
Bromeliaceae	*Tillandsia caerulea*	975	0	19	7	10	16	0	25	55	30	22	0	0	7	8	0	0	0	0	0	0	0	0	0	0	0
Bromeliaceae	*Tillandsia complanata*	1365	0	0	0	0	0	0	0	0	0	0	0	0	0	0	0	2	1	0	0	0	3	0	1	0	0
Bromeliaceae	*Tillandsia distichia*	927	39	11	18	13	29	38	26	49	40	14	67	31	9	13	8	36	16	24	4	1	0	0	0	0	0
Bromeliaceae	*Tillandsia flagellata*	923	0	0	0	0	0	0	0	0	0	0	63	15	10	17	4	298	170	177	85	99	49	23	1	0	7
Bromeliaceae	*Tillandsia floribunda*	929	7	0	0	0	0	19	3	6	0	0	0	0	0	27	0	0	0	0	2	0	154	0	1	1	12
Bromeliaceae	*Tillandsia latifolia*	916	19	9	14	17	28	0	12	11	29	4	0	0	0	0	0	0	0	0	0	0	0	0	0	0	0
Bromeliaceae	*Tillandsia recurvata*	1977	60	9	3	7	22	9	50	13	58	16	23	17	30	35	0	0	0	0	0	0	0	0	0	0	0
Bromeliaceae	*Tillandsia sp.*	3042	0	0	4	6	21	0	0	0	0	0	0	0	0	0	0	0	0	0	0	0	0	0	0	0	0
Bromeliaceae	*Tillandsia trichoglochinoides*	924	0	0	0	0	0	0	0	0	0	0	404	265	2	19	35	24	26	1	182	201	0	8	2	1	0
Bromeliaceae	*Tillandsia usneoides*	1978	9	6	4	3	0	70	73	105	14	133	22	16	200	66	149	20	1	16	0	0	0	0	1	0	2
Bromeliaceae	*Vriesea barbeyana*	930	0	0	0	0	0	0	0	0	0	0	57	31	15	19	7	34	46	28	0	0	5	0	0	0	0
Bromeliaceae	*Vriesea hitchkockiana*	3045	0	0	0	0	0	0	0	0	0	0	0	0	0	0	0	0	0	1	0	0	13	5	0	0	1
Bromeliaceae	*Vriesea spinosa*	973	55	55	68	87	116	36	29	24	34	12	209	72	20	101	36	21	22	21	0	0	0	4	1	0	0
Cactaceae	*Epiphyllus sp.*	3053	0	0	0	0	0	0	0	0	0	0	0	0	0	0	0	0	0	0	0	0	1	0	0	0	0
Cactaceae	*Hylocereus polyrhizus*	970	0	1	0	1	0	0	0	0	0	0	0	0	0	0	0	0	0	0	0	1	0	0	2	1	1
Cactaceae	*Rhipsalis micrantha*	910	0	0	0	0	0	0	0	0	0	0	3	0	0	0	0	8	7	3	9	8	7	21	7	12	13
Clusidaceae	*Clusia sp*	939	0	0	0	0	0	0	0	0	0	0	0	0	0	0	0	0	0	0	3	6	0	0	4	2	5
Dioscoreaceae	*Dioscorea sp.*	3052	0	0	0	0	0	0	0	0	0	0	0	0	0	0	0	0	0	0	0	0	6	36	0	0	1
Gesneriaceae	*Columnea sp.*	950	0	0	0	0	0	0	0	0	0	0	0	0	0	0	0	0	0	0	0	0	4	3	6	14	2
Moraceae	*Ficus jacobii*	4489	0	0	0	0	0	0	0	0	0	0	0	0	0	0	0	0	0	0	0	0	0	0	1	0	0
Moraceae	*Ficus sp. 1*	4281	0	0	0	0	0	0	0	0	1	0	0	1	0	1	0	5	2	1	1	0	0	0	0	0	1
Orchidaceae	*Anathallis pachyphyta*	937	0	0	0	0	0	0	0	0	0	0	12	0	5	0	0	1	1	0	15	14	0	0	0	0	0
Orchidaceae	*Brassia cf. warszewiciana*	945	0	0	0	0	0	0	0	0	0	0	3	4	2	0	0	0	4	0	1	0	0	0	0	0	0

Family	Species	Col. ref.	550 m					800 m					1050 m					1150 m					1250 m				
			1	2	3	4	5	1	2	3	4	5	1	2	3	4	5	1	2	3	4	5	1	2	3	4	5
Orchidaceae	*Campylocentum ecuadoriense*	933	0	0	0	0	0	0	0	0	0	0	3	1	0	0	0	1	0	3	0	0	0	1	0	0	0
Orchidaceae	*Catasetum cf. tenebrosum*	946	0	1	0	0	0	0	0	0	0	1	0	0	2	1	0	19	1	3	0	0	0	0	0	0	0
Orchidaceae	*Crocodeilanthe verbiformis*	936	0	0	0	0	0	0	0	0	0	0	0	0	0	0	0	0	0	0	14	9	13	0	0	3	7
Orchidaceae	*Encyclia aspera*	961	0	0	0	0	0	0	0	0	0	0	0	0	0	0	0	1	1	2	0	1	0	0	0	0	0
Orchidaceae	*Encyclia naranajo-patensis*	956	0	0	0	0	0	0	0	0	0	0	0	0	0	0	0	2	0	0	0	0	0	1	0	0	0
Orchidaceae	*Epidendrum aff. pichinchense*	2962	0	0	0	0	0	0	0	0	0	0	0	0	0	0	0	0	0	0	0	0	0	0	11	10	13
Orchidaceae	*Epidendrum polystachum*	935	0	0	0	0	0	0	0	0	0	0	0	0	0	0	0	0	0	0	0	0	12	16	0	0	0
Orchidaceae	*Epidendrum rimanii*	948	0	0	0	0	0	0	0	0	0	0	0	0	0	0	0	0	0	0	1	3	0	0	0	0	0
Orchidaceae	*Konanzia cf. minutiflora*	3043	0	0	0	0	0	0	0	0	0	0	0	1	0	0	0	3	5	0	0	0	0	0	0	0	0
Orchidaceae	*Maxillaria estradae*	906	0	0	0	0	0	0	0	0	0	0	0	0	0	0	0	0	0	0	4	2	14	0	0	0	1
Orchidaceae	*Maxillaria aff. multicaulis*	3054	0	0	0	0	0	0	0	0	0	0	0	0	0	0	0	0	0	0	0	1	28	0	0	0	0
Orchidaceae	*Notylia sp.*	3044	0	0	0	0	0	0	0	0	0	0	0	0	0	0	0	0	0	0	0	0	0	0	1	1	0
Orchidaceae	*Oncidium cf.*	959	0	0	0	0	0	0	0	0	0	0	0	0	0	0	0	0	0	0	0	2	19	5	0	0	0
Orchidaceae	*Oncidium sp.*	954	0	0	0	0	0	0	0	0	0	0	0	0	0	0	0	4	2	0	1	15	23	2	7	1	5
Orchidaceae	*Orchidaceae indet.*	3085	0	0	0	0	0	0	0	0	0	0	0	0	0	0	0	0	0	0	0	0	0	0	3	0	0
Orchidaceae	*Ornitocephalus dolabratus*	909	0	0	0	0	0	0	0	0	0	0	0	0	0	0	0	4	0	0	3	6	0	0	0	5	0
Orchidaceae	*Pleurothalis sp. nov.*	3051	0	0	0	0	0	0	0	0	0	0	0	0	0	0	0	0	0	0	0	0	4	0	0	0	8
Orchidaceae	*Polystachia concreta*	949	0	0	0	0	0	0	0	0	0	0	0	0	0	0	0	1	0	0	1	1	2	2	0	0	0
Orchidaceae	*Prosthechea vespa cf.*	770	0	0	0	0	0	0	0	0	0	0	0	0	0	0	0	0	0	0	0	0	0	0	1	0	1
Orchidaceae	*Psygmorchis pusilla*	908	0	0	0	0	0	0	0	0	0	0	1	0	2	0	0	0	0	0	2	0	0	0	0	0	0
Orchidaceae	*Rodriguezia refracta*	907	0	0	0	0	0	0	0	0	0	0	0	0	0	0	0	0	0	1	1	0	0	1	0	3	2
Orchidaceae	*Scaphyglottis lindeniana*	905	0	0	0	0	0	0	0	0	0	0	0	0	0	0	0	0	0	0	4	4	14	2	0	0	0
Orchidaceae	*Selencoa onusa*	958	0	0	1	0	1	0	0	0	1	0	0	0	0	0	0	0	0	0	0	0	0	0	0	0	0
Orchidaceae	*Stelis sp.*	3083	0	0	0	0	0	0	0	0	0	0	0	0	0	0	0	0	0	0	2	0	0	0	0	0	0
Orchidaceae	*Trichoncentrum trigrillum*	919	0	0	0	0	0	0	0	0	0	0	0	8	0	0	2	2	0	6	0	3	0	1	0	0	0
Piperaceae	*Peperomia galioides*	911	0	0	0	0	0	0	0	0	0	0	0	0	0	0	0	0	0	0	0	2	117	113	47	63	73
Piperaceae	*Peperomia sp. nov. 1*	913	0	0	0	0	0	0	0	0	0	0	0	0	0	0	0	0	15	0	22	25	0	0	1	0	0
Piperaceae	*Peperomia sp. nov. 2*	3048	0	0	0	0	0	0	0	0	0	0	0	0	0	0	0	0	0	0	0	0	57	2	0	0	0
Polypodiaceae	*Pleopeltis bombycina*	903	0	0	0	0	0	0	0	0	0	0	0	0	0	0	0	0	0	0	0	3	0	1	0	9	3
Polypodiaceae	*Pleopeltis polypodioides*	901	0	0	0	0	0	0	0	0	0	0	0	0	1	0	0	0	0	1	0	0	37	84	76	18	49
Polypodiaceae	*Serpocaulon attenuatum*	902	0	0	0	0	0	0	0	0	0	0	9	2	1	0	1	23	25	7	10	15	29	16	13	6	7
Polypodiaceae	*Serpocaulon lasiopus*	912	0	0	0	0	0	0	0	0	0	0	0	0	0	0	0	0	0	0	0	0	172	74	6	3	7
Urticaceae	*Pilea sp.*	3049	0	0	0	0	0	0	0	0	0	0	0	0	0	0	0	0	0	0	0	0	19	4	0	0	0

[a] Specimen collection numbers (col. no.) refer to collector Florian A. Werner except for *Ficus spp.* (Jürgen Homeier).

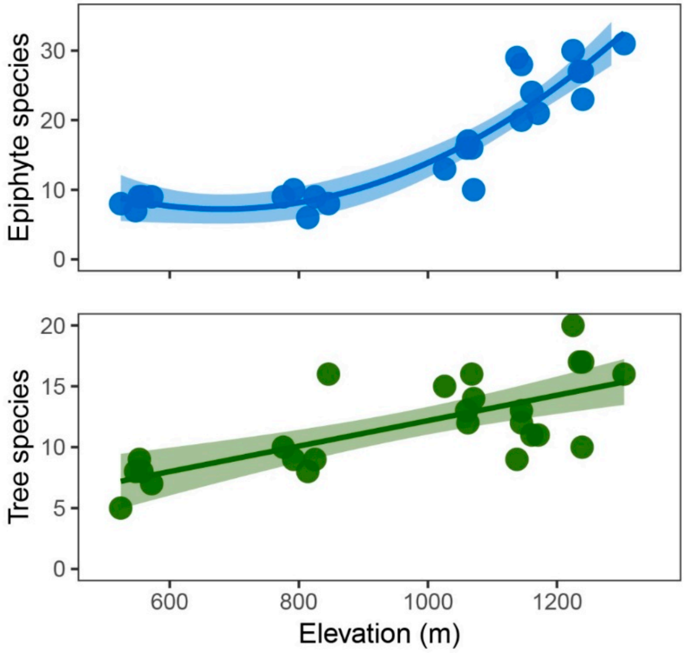

Figure S1. Raw species density (species per plot) of epiphytes (top) and trees (bottom) vs. elevation. Inserted are 2nd degree polynomial (epiphytes) and linear (trees) trendlines with 95% confidence intervals.
